# Supplementary material for: Speech and music recruit frequency-specific distributed and overlapping cortical networks
Source: eLife. 2024 Jul 22;13:RP94509. doi: 10.7554/eLife.94509 (PMC11262799; doi:10.7554/eLife.94509)
Supplement: Supplementary file 1. — Table provides an overview of patient characteristics and experimental conditions. The table includes 18 patients, with a mix of males and females aged between 8 and 54 years (mean 30). Presentation order (either speech-music or music-speech) was counterbalanced between patients. Recordings took place either at the bedside (room) or in the lab. Hemispheric dominance was mostly typical. All patients had an electrode implanted in the auditory cortex (Heschl’s gyrus; left, right, or bilaterally). The number of depth electrodes is indicated in the far right column. [file elife-94509-supp1.docx]

| **Patient** | **Gender** | **Age** | **Order of Presentation** | **Recording Site** | **Hemispheric Dominance (Typical/Atypical)** | **H: Left/Right** | **Depth Electrodes** |
| --- | --- | --- | --- | --- | --- | --- | --- |
| P1 | f | 35 | speech-music | room | Typical | Bilateral | 11 left; 3 right |
| P2 | m | 29 | speech-music | room | AtypicalB | Left | 12 left; 2 right |
| P3 | m | 39 | speech-music | lab | Typical | Left | 14 left; 2 right |
| P4 | m | 8 | speech-music | lab | Typical | Bilateral | 10 left; 2 right |
| P5 | f | 36 | speech-music | room | Typical | Left | 13 left; 3 right |
| P6 | m | 44 | music-speech | room | Typical | Left | 15 left; 2 right |
| P7 | f | 37 | speech-music | lab | AtypicalB | Bilateral | 7 left; 9 right |
| P8 | f | 17 | speech-music | lab | Typical | Bilateral | 11 left; 4 right |
| P9 | f | 54 | music-speech | room | Typical | Left | 15 left; 5 right |
| P10 | f | 20 | music-speech | lab | Typical | Right | 1 left; 11 right |
| P11 | f | 37 | music-speech | room | Typical | Left | 11 left; 2 right |
| P12 | f | 37 | music-speech | room | Typical | Left | 14 left; 2 right |
| P13 | m | 24 | music-speech | room | AtypicalB | Left | 13 left; 2 right |
| P14 | f | 30 | music-speech | room | Typical | Left | 12 left; 1 right |
| P15 | f | 22 | music-speech | room | Typical | Right | 1 left; 11 right |
| P16 | m | 22 | music-speech | room | Typical | Bilateral | 12 left; 3 right |
| P17 | m | 32 | music-speech | room | Typical | Left | 10 left; 2 right |
| P18 | m | 17 | speech-music | lab | Typical | Right | 14 left; 1 right |

***Supplementary Table 1: Patients description.*** *Table provides an overview of patient characteristics and experimental conditions. The table includes 18 patients, with a mix of males and females aged between 8 and 54 years (mean 30). Presentation order (either speech-music or music-speech) was counterbalanced between patients. Recordings took place either at the bedside (room) or in the lab. Hemispheric dominance was mostly typical. All patients had an electrode implanted in the auditory cortex (Heschl gyrus; left, right, or bilaterally). The number of depth electrodes is indicated in the far right column.*
