# Supplementary material for: Speech and music recruit frequency-specific distributed and overlapping cortical networks
Source: eLife. 2024 Jul 22;13:RP94509. doi: 10.7554/eLife.94509 (PMC11262799; doi:10.7554/eLife.94509)
Supplement: MDAR checklist [file elife-94509-mdarchecklist1.docx]

Materials Design Analysis Reporting (MDAR)

Checklist for Authors

The [MDAR framework](https://osf.io/xfpn4/) establishes a minimum set of requirements in transparent reporting mainly applicable to studies in the life sciences.

*eLife* asks authors to provide detailed information within their article to facilitate the interpretation and replication of their work. Authors can also upload supporting materials to comply with relevant reporting guidelines for health-related research (see [EQUATOR Network](http://www.equator-network.org/%20)), life science research (see the [BioSharing Information Resource](http://biosharing.org/)), or animal research (see the [ARRIVE Guidelines](http://www.plosbiology.org/article/info:doi/10.1371/journal.pbio.1000412) and the [STRANGE Framework](https://doi.org/10.1038/d41586-020-01751-5); for details, see *eLife*’s [Journal Policies](https://reviewer.elifesciences.org/author-guide/journal-policies)). Where applicable, authors should refer to any relevant reporting standards materials in this form.

For all that apply, please note where in the article the information is provided. Please note that we also collect information about data availability and ethics in the submission form.

Materials:

| Newly created materials | Indicate where provided: section/figure legend | N/A |
| --- | --- | --- |
| The manuscript includes a dedicated "materials availability statement" providing transparent disclosure about availability of newly created materials including details on how materials can be accessed and describing any restrictions on access. |  | x |
|  |  |  |
| Antibodies | Indicate where provided: section/figure legend | N/A |
| For commercial reagents, provide supplier name, catalogue number and [RRID](https://scicrunch.org/resources), if available. |  | x |
|  |  |  |
| DNA and RNA sequences | Indicate where provided: section/figure legend | N/A |
| Short novel DNA or RNA including primers, probes: Sequences should be included or deposited in a public repository. |  | x |
|  |  |  |
| Cell materials | Indicate where provided: section/figure legend | N/A |
| Cell lines: Provide species information, strain. Provide accession number in repository OR supplier name, catalog number, clone number, OR RRID. |  | x |
| Primary cultures: Provide species, strain, sex of origin, genetic modification status. |  | x |
|  |  |  |
| Experimental animals | Indicate where provided: section/figure legend | N/A |
| Laboratory animals or Model organisms: Provide species, strain, sex, age, genetic modification status. Provide accession number in repository OR supplier name, catalog number, clone number, OR RRID. |  | x |
| Animal observed in or captured from the field: Provide species, sex, and age where possible. |  | x |
|  |  |  |
| Plants and microbes | Indicate where provided: section/figure legend | N/A |
| Plants: provide species and strain, ecotype and cultivar where relevant, unique accession number if available, and source (including location for collected wild specimens). |  | x |
| Microbes: provide species and strain, unique accession number if available, and source. |  | x |
|  |  |  |
| Human research participants | Indicate where provided: section/figure legend) or state if these demographics were not collected | N/A |
| If collected and within the bounds of privacy constraints report on age, sex, gender and ethnicity for all study participants. | The study reports data collected from 18 patients: 10 females, mean age 30 y, range 8 - 54 y. All patients were French native speakers. See Supplementary Table 1 for more information. |  |

Design:

| Study protocol | Indicate where provided: section/figure legend | N/A |
| --- | --- | --- |
| If the study protocol has been pre-registered, provide DOI. For clinical trials, provide the trial registration number OR cite DOI. |  | x |
|  |  |  |
| Laboratory protocol | Indicate where provided: section/figure legend | N/A |
| Provide DOI OR other citation details if detailed step-by-step protocols are available. |  | x |
|  |  |  |
| Experimental study design (statistics details) * | | |
| For in vivo studies: State whether and how the following have been done | Indicate where provided: section/figure legend. If it could have been done, but was not, write “not done” | N/A |
| Sample size determination |  | x |
| Randomisation |  | x |
| Blinding |  | x |
| Inclusion/exclusion criteria |  | x |
|  |  |  |
| Sample definition and in-laboratory replication | Indicate where provided: section/figure legend | N/A |
| State number of times the experiment was replicated in the laboratory. |  | x |
| Define whether data describe technical or biological replicates. |  | x |
|  |  |  |
| Ethics | Indicate where provided: section/submission form | N/A |
| Studies involving human participants: State details of authority granting ethics approval (IRB or equivalent committee(s), provide reference number for approval. | Patients provided informed consent prior to the experimental session, and the experimental protocol was approved by the Institutional Review board of the French Institute of Health (IRB00003888). |  |
| Studies involving experimental animals: State details of authority granting ethics approval (IRB or equivalent committee(s), provide reference number for approval. |  | x |
| Studies involving specimen and field samples: State if relevant permits obtained, provide details of authority approving study; if none were required, explain why. |  | x |
|  |  |  |
| Dual Use Research of Concern (DURC) | Indicate where provided: section/submission form | N/A |
| If study is subject to dual use research of concern regulations, state the authority granting approval and reference number for the regulatory approval. |  | x |

Analysis:

| Attrition | Indicate where provided: section/figure legend | N/A |
| --- | --- | --- |
| Describe whether exclusion criteria were pre-established. Report if sample or data points were omitted from analysis. If yes, report if this was due to attrition or intentional exclusion and provide justification. | Artifacted channels and epochs were excluded from the analyzes. To define artifacted channel we used both the broadband signal and the amplitude of the high frequency activity. Channels with a variance greater than 2*IQR (interquartile range, i.e. a non-parametric estimate of the standard deviation)—on either the broadband or high frequency signals—were tagged as artifacted channels (on average 18% of the channels). Then the data were epoched in non-overlapping segments of 5 seconds (2500 samples). To exclude artifacted epochs, epochs wherein the maximum amplitude (over time) summed across non-excluded channels was greater than 2*IQR were tagged as artifacted epochs. Overall, 6% of the speech epochs and 7% of the music epochs were rejected. |  |
|  |  |  |
| Statistics | Indicate where provided: section/figure legend | N/A |
| Describe statistical tests used and justify choice of tests. | **Spectral analysis.**  For each canonical band and each channel, we classified the time-averaged neural response as being selective, preferred, or shared across the two investigated cognitive domains (speech, music). We defined these categories by capitalizing on both the simple effects of—and contrast between—the neural responses to speech and music stimuli compared to a baseline condition (see Figure 1A). For each frequency band and channel, the statistical difference between conditions was estimated with paired sample permutation tests based on the t-statistic from the mne-python library (Gramfort et al., 2014) with 1000 permutations and the *tmax* method to control the family-wise error rate [(Groppe et al., 2011; Nichols & Holmes, 2002)](https://paperpile.com/c/iM5zul/1oVD+6PpQ). In *tmax* permutation testing, the null distribution is estimated by, for each channel (i.e. each comparison), swapping the condition labels (speech vs music or speech/music vs baseline) between epochs. After each permutation, the most extreme t-scores over channels (*tmax*) are selected for the null distribution. Finally, the t-scores of the observed data are computed and compared to the simulated *tmax* distribution, similar as in parametric hypothesis testing. Because with an increased number of comparisons, the chance of obtaining a large *tmax* (i.e. false discovery) also increases, the test automatically becomes more conservative when making more comparisons, as such correcting for the multiple comparison between channels.  **Temporal Response Function (TRF) analysis.**  We used the Temporal Response Function (TRF) to estimate the encoding of acoustic features by neural activity. The quality of the predicted neural response was assessed by computing Pearson’s product moment correlations (Fisher-z-scored) between the predicted and actual neural data for each channel and model using the scipy-python library (p-values FDR-corrected).  Models were finally compared in terms of the percentage of channels that significantly encoded the acoustic structure of speech and/or music. This percentage was estimated at the single-subject level and combined with non-parametric Wilcoxon sign rank tests at the group level to define the winning model. In other words, the winning model is the model for which the percentage of channels significantly encoding speech and/or music acoustic features is the largest. Multiple comparison across pairs of models was controlled for with a FDR correction.  **Connectivity analysis.**  We examined the frequency-specific functional connectivity maps in response to speech and music, between the entire brain and the auditory cortex using a seed-based approach. Statistical significance was assessed for each frequency band and channel using surrogate data with 1000 iterations, which were generated by modifying the temporal structure of the sEEG-signal recorded at the seeds (i.e. shuffling the epochs) prior to computing connectivity. This process led to a total of 1000 connectivity values, which were used as null-distribution to calculate the probability threshold associated with genuine connectivity. |  |
|  |  |  |
| Data availability | Indicate where provided: section/submission form | N/A |
| For newly created and reused datasets, the manuscript includes a data availability statement that provides details for access (or notes restrictions on access). | The raw data investigated in the current manuscript is privileged patient data. Because of this, the conditions of our ethics approval do not permit public archiving of anonymised study data. Readers seeking access to the data should contact Dr. Daniele Schön (daniele.schon@univ-amu.fr). Access will be granted to named individuals in accordance with ethical procedures governing the reuse of clinical data, including completion of a formal data sharing agreement. Preprocessed data necessary to reproduce the figures and results are available on Github: github.com/noemietr/iSpeech |  |
| When newly created datasets are publicly available, provide accession number in repository OR DOI and licensing details where available. |  | x |
| If reused data is publicly available provide accession number in repository OR DOI, OR URL, OR citation. |  | x |
|  |  |  |
| Code availability | Indicate where provided: section/figure legend | N/A |
| For any computer code/software/mathematical algorithms essential for replicating the main findings of the study, whether newly generated or re-used, the manuscript includes a data availability statement that provides details for access or notes restrictions. | yes: Data analyses were performed using custom scripts in Python, available on Github: github.com/noemietr/iSpeech |  |
| Where newly generated code is publicly available, provide accession number in repository, OR DOI OR URL and licensing details where available. State any restrictions on code availability or accessibility. | Data analyses were performed using custom scripts in Python, available on Github: github.com/noemietr/iSpeech |  |
| If reused code is publicly available provide accession number in repository OR DOI OR URL, OR citation. |  | x |

Reporting:

The MDAR framework recommends adoption of discipline-specific guidelines, established and endorsed through community initiatives.

| Adherence to community standards | Indicate where provided: section/figure legend | N/A |
| --- | --- | --- |
| State if relevant guidelines (e.g., ICMJE, MIBBI, ARRIVE, STRANGE) have been followed, and whether a checklist (e.g., CONSORT, PRISMA, ARRIVE) is provided with the manuscript. |  | x |

* We provide the following guidance regarding transparent reporting and statistics; we also refer authors to [Ten common statistical mistakes to watch out for when writing or reviewing a manuscript](https://doi.org/10.7554/eLife.48175).

Sample-size estimation

- You should state whether an appropriate sample size was computed when the study was being designed
- You should state the statistical method of sample size computation and any required assumptions
- If no explicit power analysis was used, you should describe how you decided what sample (replicate) size (number) to use

Replicates

- You should report how often each experiment was performed
- You should include a definition of biological versus technical replication
- The data obtained should be provided and sufficient information should be provided to indicate the number of independent biological and/or technical replicates
- If you encountered any outliers, you should describe how these were handled
- Criteria for exclusion/inclusion of data should be clearly stated
- High-throughput sequence data should be uploaded before submission, with a private link for reviewers provided (these are available from both GEO and ArrayExpress)

Statistical reporting

- Statistical analysis methods should be described and justified
- Raw data should be presented in figures whenever informative to do so (typically when N per group is less than 10)
- For each experiment, you should identify the statistical tests used, exact values of N, definitions of center, methods of multiple test correction, and dispersion and precision measures (e.g., mean, median, SD, SEM, confidence intervals; and, for the major substantive results, a measure of effect size (e.g., Pearson's r, Cohen's d)
- Report exact p-values wherever possible alongside the summary statistics and 95% confidence intervals. These should be reported for all key questions and not only when the p-value is less than 0.05.

Group allocation

- Indicate how samples were allocated into experimental groups (in the case of clinical studies, please specify allocation to treatment method); if randomization was used, please also state if restricted randomization was applied
- Indicate if masking was used during group allocation, data collection and/or data analysis
